# Ohio State Dental Society

**Published:** 1867-06

**Authors:** A. W. Maxwell

**Affiliations:** Galion


### OHIO STATE DENTAL SOCIETY.

The Semi-Annual Meeting of the "Ohio State Dental Society" will convene in the City of Columbus, at 10 o'clock, A. M., on Tuesday, June 18, 1867. The following subjects have been selected by the Executive Committee for discussion:

1. The different preparations of Gold for filling Teeth.
2. Filling over exposed Pulps.
3. Correcting irregularities of Teeth.
4. Necrosis; causes and treatment.
5. Professional standing of the Dentist.
6. Miscellaneous.

You are cordially invited to be present at this meeting, and to bring your friends in the profession with you.

GEO. WATT, Pres't, A. W. MAXWELL, Cor. Sec'y,
*Galion.*

